# Effects of Natural Polyphenols on Oxidative Stress-Mediated Blood-Brain Barrier Dysfunction

**DOI:** 10.3390/antiox11020197

**Published:** 2022-01-20

**Authors:** Yeonjae Kim, A Yeon Cho, Hong Cheol Kim, Dajung Ryu, Sangmee Ahn Jo, Yi-Sook Jung

**Affiliations:** 1 College of Pharmacy, Ajou University, Suwon 16499, Korea; yeonjai95@ajou.ac.kr (Y.K.); zinci@ajou.ac.kr (A.Y.C.); rgo522@ajou.ac.kr (H.C.K.); cbrdj0511@ajou.ac.kr (D.R.); 2 Research Institute of Pharmaceutical Sciences and Technology, Ajou University, Suwon 16499, Korea; 3 Department of Nanobiomedical Science & BK21 NBM Global Research Center for Regenerative Medicine, Dankook University, Cheonan 31116, Korea; smahn@dankook.ac.kr; 4 Department of Pharmacology, College of Pharmacy, Dankook University, Cheonan 31116, Korea

**Keywords:** blood-brain barrier (BBB), oxidative stress, reactive oxygen species (ROS), natural polyphenols

## Abstract

The blood-brain barrier (BBB), which consists mainly of brain microvascular endothelial cells and astrocytes connected by tight junctions (TJs) and adhesion molecules (AMs), maintains the homeostatic balance between brain parenchyma and extracellular fluid. Accumulating evidence shows that BBB dysfunction is a common feature of neurodegenerative diseases, including stroke, traumatic brain injury, and Alzheimer’s disease. Among the various pathological pathways of BBB dysfunction, reactive oxygen species (ROS) are known to play a key role in inducing BBB disruption mediated via TJ modification, AM induction, cytoskeletal reorganization, and matrix metalloproteinase activation. Thus, antioxidants have been suggested to exert beneficial effects on BBB dysfunction-associated brain diseases. In this review, we summarized the sources of ROS production in multiple cells that constitute or surround the BBB, such as BBB endothelial cells, astrocytes, microglia, and neutrophils. We also reviewed various pathological mechanisms by which BBB disruption is caused by ROS in these cells. Finally, we summarized the effects of various natural polyphenols on BBB dysfunction to suggest a therapeutic strategy for BBB disruption-related brain diseases.

## 1. Introduction

The blood-brain barrier (BBB) is a structural membranous barrier that restricts the passage of molecules circulating in the blood into the brain and functions to maintain the homeostatic balance of the extracellular fluid in the brain under physiological conditions [1]. The BBB primarily comprises brain microvascular endothelial cells (ECs), astrocytes, and pericytes, which are characterized by a few pinocytic vesicles, abundant mitochondria, interendothelial tight junctions (TJs), and adherens junctions (AJs) [1]. While the low pinocytic activity of the BBB limits transcellular transport of molecules across it, paracellular permeability of the BBB can be regulated by TJ proteins, such as claudin and occludin, and AJ proteins, such as cadherin and catenin [2,3]. Occludin and claudins are known to be anchored to BBB endothelial cells by scaffolding proteins which consist of a family of zonula occludens (ZO) proteins, such as ZO-1, ZO-2, and ZO-3 [4]. The expression of TJ proteins and AJ proteins, such as claudin and cadherin, respectively, is closely associated with the permeability of the BBB. Indeed, increased expression of occludin has been shown to decrease permeability across the BBB by inducing a decrease in paracellular transport [5], and increased claudin-5 expression results in the reduction of the transit of large molecules [6].

Under pathological conditions, disruption of the BBB is common and plays a critical role in the pathological process of various cerebrovascular diseases, including stroke and Alzheimer’s disease (AD) [7,8]. Reactive oxygen species (ROS) play a major causative role in the disruption of the BBB by triggering signaling pathways that mediate the breakdown of TJ proteins and modification of AJ proteins. Accumulating evidence further demonstrates the role of ROS in the activation of matrix metalloproteinases (MMPs), a group of proteolytic enzymes that can degrade extracellular matrix components, leading to the disruption of the BBB [1,9]. In this context, it is suggested that antioxidants can act as potential neuroprotectants under disease conditions that induce damage to the BBB.

In the last decade, natural antioxidants have garnered significant interest in the development of promising therapeutics because of their safety, convenience, and bioactivities [10]. The most abundant types of these antioxidants are carotenoids (xanthophylls and carotenes), vitamins (vitamin E and C), and polyphenols (phenolic acids, flavonoids, anthocyanins, lignans, and stilbenes) [11,12]. Compared to carotenoids, the main scavengers of the ROS, such as singlet oxygen and peroxy radicals [13,14], and vitamin C, a chain-disrupting scavenger of peroxy radicals [15]. Polyphenols are the most numerous and widely distributed bioactive molecules, and predominantly contribute to the total antioxidant properties exhibited by various food types [16]. Polyphenols are advantageous, as they exhibit various antioxidant activities, such as inhibition of ROS generation, inactivation of ROS precursors, metal chelation, and ROS scavenging. Furthermore, polyphenols have been shown to exert beneficial effects against pathological conditions, such as cancer [17], type-2 diabetes mellitus, cardiovascular diseases, and cerebrovascular diseases [18,19,20].

In this review, we first summarize the ROS-generating pathways in the multiple cells that make up or surround the BBB, such as BBB ECs, astrocytes, microglia, and neutrophils. We also review the pathological mechanisms underlying BBB disruption is induced by ROS in those cells. We then provide an overview of the effects of various natural polyphenols on BBB dysfunction to suggest a therapeutic strategy for BBB-associated neurodegenerative diseases.

## 2. Oxidative Stress in Multiple Cells That Constitute or Surround the BBB

Under normal physiologic conditions, low levels of ROS formation within vascular cells are well controlled by the endogenous antioxidant system, which comprises important signaling molecules for normal vascular function. Increasing evidence demonstrates that the physiological impact of ROS depends on their intracellular levels as well as their chemical nature and subcellular localization [21,22]. Therefore, inappropriate scavenging of ROS may induce paradoxical reductive stress and, thereby, cause pathological disease [23,24,25]. Moreover, oxidative stress can be observed upon an imbalance between ROS formation and ROS removal, and plays a critical role in the pathogenesis of various diseases, including atherosclerosis, inflammatory diseases, and neurodegenerative disorders [26]. Under pathological conditions in BBB, excess ROS can activate a number of signaling molecules, such as hypoxia-inducible factor-1, nuclear factor kappa-light-chain-enhancer of activated B cells (NF-κB), and MMPs, which result in loss of BBB integrity and BBB dysfunction.

The cells surrounding the BBB, including BBB ECs and astrocytes, are rich in mitochondria, one of the main sources of ROS, especially under pathological conditions. Although mitochondrial ROS have recently been suggested to be involved in cerebrovascular diseases [27], little is known about their roles in individual cells during the process of BBB disruption. In contrast, many studies have examined other ROS sources, including nitric oxide synthase (NOS) and nicotinamide adenine dinucleotide phosphate (NADPH) oxidase (NOX) [28,29]. Therefore, this chapter summarizes the sources of ROS generation in each BBB surrounding cell by focusing on NOX, NOS, xanthine oxidase (XO), and cyclooxygenase (COX) rather than mitochondrial ROS.

### 2.1. Oxidative Stress in BBB Endothelial Cells during BBB Injury

**Figure 1:**
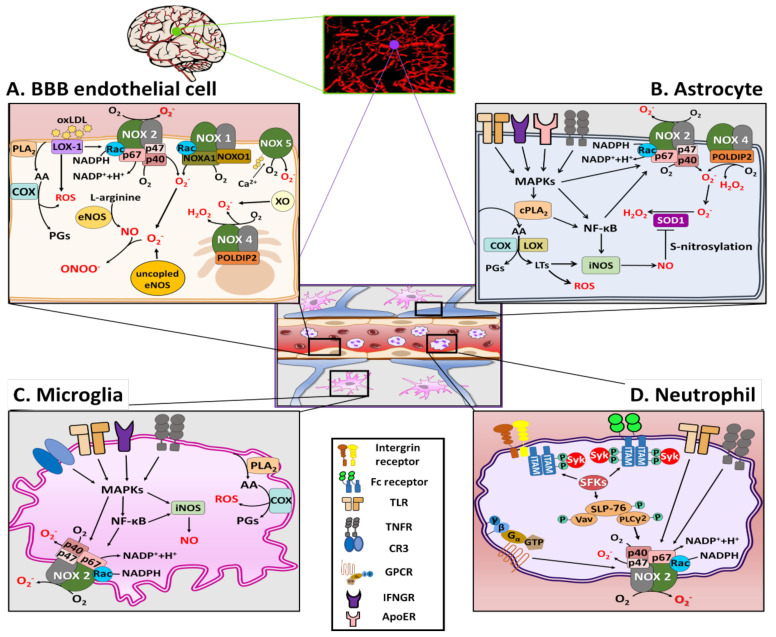
Schematic illustration of ROS production in multiple cells that constitute or surround the BBB. (**A**) ROS production in BBB ECs. BBB ECs generate ROS via XO, COX, NOS, and NOX. The NOX family primarily reduces O_2_ to O_2_^−^. COX generates ROS by catalyzing the conversion of AA to PGs. LOX-1 activates NOX2 through the binding of oxLDL, resulting in the production of ROS. Under pathophysiological conditions, eNOS can produce ONOO^−^ with L-arginine and also generate O_2_^−^ via conversion to uncoupled eNOS. (**B**) ROS production in astrocytes. Under a pathophysiological condition, increased activity of NOX2 and NOX4 can result in the generation of O_2_^−^ and H_2_O_2_. ApoE4, LPS, and IFN induce cPLA_2_ activation via p38MAPK, leading to iNOS activation and ROS generation. Increased expression of iNOS results in the generation of NO, with subsequent aggregation of SOD1 via S-nitrosylation of protein disulfide isomerase. (**C**) ROS production in microglia. Microglia generate ROS primarily by NOX2. DAMP mediated stimulation of PRRs and pro-inflammatory stimuli can activate MAPKs and NF-κB, resulting in NOX2 activation. Microglia also generate NO via iNOS. The expression of COX in microglia can mediate ROS generation by catalyzing the conversion of AA to PGs. (**D**) ROS production in neutrophils. Under pathophysiological condition, neutrophil generates ROS primarily by NOX2. Ligation of FcγRs phosphorylates ITAM by SFKs resulting in activation of Syk, which induces SLP76 activation, leading to the activation of NOX2. AA, arachidonic acid; ApoE, Apolipoprotein E; BBB EC, blood-brain barrier endothelial cell; COX, cyclooxygenase; CR3, complement receptor3; cPLA_2_, cytosolic PLA_2_ DAMPs: damage-associated molecular patterns; eNOS: endothelial NOS; FcγRs, Fcγ receptors; GPCRs, G protein-coupled receptors; IFN, interferon; iNOS, inducible NOS; ITAM, immunoreceptor tyrosine-based activation motif; LOX, lipoxygenase; LOX-1, lectin-like oxidized low-density lipoprotein receptor-1; LPS, lipopolysaccharide; MAPK, mitogen-activated protein kinase; NADPH, nicotinamide adenine dinucleotide phosphate; NF-kB, nuclear factor kappa-light-chain-enhancer of activated B cells; NOS, nitric oxide synthase; NOX, NADPH oxidase; oxLDL, oxidized low-density lipoprotein; p40, p40^phox^; p47, p47^phox^; p67, p67^phox^; PGs, bioactive prostaglandins; PLA_2_, phospholipase A2; PLCγ2, phospholipase C γ2; PRRs, pattern recognition receptors; SFK, Src family kinase; SLP76, SH2-domain- containing leukocyte protein of 76 kDa; SOD1, superoxide dismutase; Syk, spleen tyrosine kinase; TLR4, Toll-like receptor 4; TNFR, TNF receptor; XO, xanthine oxidase.

As shown in Figure 1A, there are several sources of ROS generation in BBB ECs, including NOS, XO, COX, and NOX family [28,29]. Among these, NOXs are known to be the primary source of ROS in the BBB ECs [30]. To date, seven isoforms of NOX have been identified: NOX1, NOX2, NOX3, NOX4, NOX5, DUOX1, and DUOX2. In general, all activated NOX isoforms can generate superoxide (O_2_^−^) by transferring electrons from the substrate NADPH via the catalytic subunit to molecular oxygen (O_2_) to form NADP^+^ and H^+^. Recent research provides a new perspective on the importance of the NOX family, particularly NOX1 and NOX2, in the generation of oxidative stress in BBB ECs [31,32]. Along with NOX1 and NOX2, NOX4 has also been reported as a major source of O_2_^−^, which induces apoptosis of BBB ECs exposed to the pro-inflammatory cytokines [33]. However, NOX4 preferentially generates H_2_O_2_ rather than O_2_^−^ [34]. Accumulating evidence also shows that under condition of ischemia/reperfusion (I/R), increased levels of intracellular Ca^2+^ result in activation of NOX5 and subsequently increases the production of O_2_^−^, OONO^−^, and H_2_O_2_, leading to the disruption of the BBB [35,36]. Consistently, levels of ROS were increased following I/R injury in hippocampal brain slices from NOX-5 knock-in mice [36]. Some studies have shown that NOX inhibitors can improve the barrier function of the BBB in several disease models [37].

NOS is another possible source of ROS production. The NOS family consist of three members: neuronal NOS (nNOS), endothelial NOS (eNOS), both of which are constitutively expressed Ca^2+^-dependent enzymes, and inducible NOS (iNOS), whose expression can be induced in a Ca^2+^-independent manner [38]. Under pathophysiological conditions, aberrant eNOS expression can contribute to the development of vascular disease via increased oxidative stress (ONOO^−^) and conversion of eNOS to an O_2_^−^-producing enzyme (uncoupled eNOS), which further generates O_2_^−^ rather than NO because of the decreased bioavailability of the tetrahydrobiopterin (BH4, cofactor) [38,39,40]. Hence, aberrant eNOS expression in BBB ECs is likely to account for increased protein tyrosine nitration by ONOO^−^, thereby inducing BBB disruption [40].

Furthermore, lectin-like low-density lipoprotein oxLDL receptor (LOX-1) is another possible source of ROS production in BBB ECs. LOX-1 regulates ROS production by influencing NOX activity in ECs. The binding of LOX-1 to ox-LDL significantly increases NOX activity via induction of *NOX-2* and *NOX-4* mRNA expression [41,42]. LOX-1 activation induced by oxLDL not only causes membrane translocation of p47^phox^ and Rac, but also protein expression of gp91 and p22^phox^ of NOX-2, which is mediated through protein kinase C (PKC) activation [43]. LOX-1 activation can also lead to the generation of ONOO^−^, causing BBB disruption [44].

COXs and XOs are also possible sources of ROS generation in BBB ECs. COXs are heme-containing enzymes that catalyze the conversion of arachidonic acid (AA) to prostaglandins (PGs), which are also important source of ROS in the brain and BBB ECs [45]. There are two main isoforms of COX: the COX-1 isoform is a housekeeping enzyme constitutively expressed in all tissues, and COX-2 can be expressed to cause inflammation, particularly expressed in the renal medulla and renal pelvis, the gastrointestinal tract, lung, thymus, and brain [46]. A recent study showed that angiotensin II (AII) increased ROS generation in bEnd3 cells via upregulation of COX-2 expression [47]. XO also generates ROS after oxygen deprivation in BBB ECs [48]. In a rat stroke model, XO inhibitor allopurinol decreased the infarct size, possibly through an antioxidant effect [49,50].

### 2.2. Oxidative Stress in Astrocytes during BBB Injury

Astrocytes play a vital role in maintaining the physiological functions of the central nervous system (CNS), such as nourishing neurons, maintaining BBB integrity, regulating synaptic activity, and processing cellular metabolites [51]. Physiologically, astrocytes can protect the CNS from oxidative stress-induced damage by exerting antioxidant effect, whereas they act as one of the main sources of detrimental ROS and reactive nitrogen species (RNS) under pathological conditions [52,53]. During brain injury, the state of astrocytes is altered from resting to reactive, and reactive astrocytes exert both protective and detrimental functions [54].

Among the NOXs, NOX2 and NOX4 are major isoforms responsible for the production of NOX-derived ROS in astrocytes [55]. In an AD model, amyloid-β (Aβ) could increase NOX2 activity and O_2_^−^ levels in astrocytes, thereby inducing astrogliosis [56]. In rat brain, astrocyte-1 (RBA-1) cells activate NOX2 and increase ROS generation [57]. Activated cytosolic phospholipase A_2_ (cPLA_2_) in astrocytes is reported to interact with the mitochondrial antiviral-signaling protein and activate NF-κB, which may be involved in inducing the expression NOXs, thereby leading to ROS generation [58,59]. An in vitro study on astrocytes demonstrated that human immunodeficiency virus-1 glycoprotein 120-induced apoptotic cell death by mediating oxidative stress via NOX2 and NOX4 [60]. In an in vitro hypoosmotic swelling model, NOX2-mediated ROS production was increased in astrocytes [61], suggesting a causative role of ROS in astrocyte swelling [62].

Astrocytes also express iNOS, and the RNS generated by them are also key a component of astrocyte-induced oxidative stress [63,64]. In an in vitro astrocyte culture study, lipopolysaccharide (LPS) was shown to induce iNOS expression, leading to NO production [65]. Brain injury induced by severe systemic inflammation is associated with the NF-κB-mediated activation of iNOS in astrocytes [66]. Moreover, in an in vitro I/R model of astrocytes, NO produced by iNOS resulted in aggregation of superoxide dismutase 1 (SOD1) via *S*-nitrosylation of protein disulfide isomerase, which may be related to the pathogenesis of the injury [67]. Recently, ApoE4 has been reported to induce cPLA_2_ activation via the p38MAPK pathway followed by leukotriene B_4_ production via lipoxygenase activation, leading to iNOS activation and ROS generation, which results in oxidative stress and neuroinflammation [68]. Taken together, oxidative stress produced by NOXs and iNOS in astrocytes appear to play a crucial role in BBB injury, as shown in Figure 1B.

### 2.3. Oxidative Stress in Microglia during BBB Injury

Microglia, the brain-resident macrophages, are known to be dynamic mediators of cerebrovascular diseases. These phagocytic glial cells form a complex network that can respond toward damage and pathogen-associated stimuli, mediating either protective or deleterious responses to brain injury [69,70]. Microglia respond to damage-associated molecular patterns (DAMPs), molecules released from damaged neurons. This results in the activation of disease-associated microglia (DAM) [70]. There is a strong correlation between microglial activation and oxidative stress, ultimately leading to neurovascular injury [71,72].

**Figure 2:**
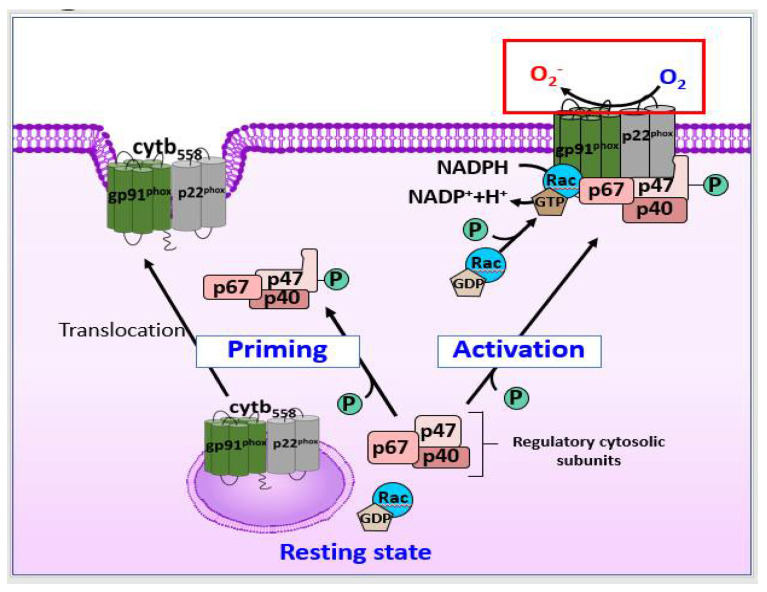
Schematic illustration of NOX2 in priming and activation state. NOX2 is a multi-protein electron transfer system that is made up five components, i.e., gp91^phox^, p22^phox^, p40^phox^, p47^phox^, and p67^phox^. In the resting state, the membrane-bound catalytic core of NOX2, composed of gp91^phox^ and p22^phox^, forms a cytb_558_, and the regulatory trimeric complex which is composed of p40^phox^, p47^phox^, and p67^phox^ resides in the cytosol of cells. Priming state induces translocation of cytb_558_ to the plasma membrane via granule exocytosis and partial phosphorylation of p47^phox^, leading to conformational changes. Upon activation, Rac exchanges GDP to GTP for direct binding to p67^phox^ and gp91^phox^. The regulatory cytosolic subunits translocate to the membranes and binds to cytb_588_, leading to NOX activation. cyt_b558_, flavocytochrome b558; GDP, guanosine diphosphate; GTP, Guanosine triphosphate; NADP/NADPH, nicotinamide adenine dinucleotide phosphate; NOX, NADPH oxidase; phox, phagocyte oxidase; p40, p40^phox^; p47, p47^phox^; p67, p67^phox^.

As shown in Figure 1C, in microglia, ROS that induce BBB dysfunction are primarily generated by NOX2, and the activation of NOX2 in DAM is associated with DAMPs, such as Aβ, high mobility group box 1 (HMGB1), and fibrinogen, during both acute and chronic neuroinflammation [73,74]. DAMP stimulation of pattern recognition receptors on the microglia, such as complement receptor 3 and Toll-like receptor 4 (TLR4), mediates activation of the NLRP3 (nucleotide-binding oligomerization domain leucine rich repeat and pyrin domain-containing protein 3) inflammasome, along with that of NF-κB and mitogen-activated protein kinases (MAPKs), such as the extracellular signal-regulated kinases (ERK) [75,76,77]. As shown in Figure 2, upon microglial activation, signaling via pro-inflammatory stimuli mediated by interferon (IFN)-γ results in the activation of cytosolic regulatory subunits of NOX2, resulting in their translocation to the membrane to assemble the active enzyme complex with flavocytochrome b558 (cyt_b558_) [78]. Serine-threonine kinases, including the MAPK family and protein kinase C, are also capable of catalyzing NOX2 assembly. The rate-limiting step of NOX2 activation involves serine-threonine phosphorylation of p47^phox^ by p21 (cdc42/Rac1)-activated kinase-1 [79]. The activated small GTPase Rac1/2 can also translocate to the membrane subunit, cyt_b558_.

Microglia can also generate RNS via NOS. Activation of microglia includes its polarization, which can lead to persistent RNS production via expression of iNOS [80,81], subsequently inducing damage to healthy host cells [82]. In addition, crosstalk between the pathways of NOS and NOX has been demonstrated. ONOO^−^ is an RNS family originated principally by the enzyme NOX through the reaction of NO and O_2_^−^. These NOs are generated via stimuli produced by LPS, IFN-γ, tumor necrosis factor (TNF)-α, interleukin (IL)-1β, arachidonate, and ATP in microglia [83]. LPS and IFN-γ lead to the activation of cPLA_2_ and MAPKs, and thereafter, activation of iNOS and NOX, resulting in the production of ROS and NO [84].

Recent studies have shown that the expression of prostaglandin-endoperoxide synthase 2 (PTGS2), also known as COX2, is highly upregulated in response to LPS and is associated with ROS in microglia [85]. PTGS2 plays a very important role in arachidonic acid metabolism, codifying COX2 enzymes, and inducing PG production. COX2 is rapidly expressed in response to various pro-inflammatory cytokines, such as IL-1β, IL-6, and TNF-α, and thereafter produces PGE2 and free radicals. PGE2, in turn, adversely affects the brain by inducing inflammation, oxidative stress, and excitotoxicity [86].

### 2.4. Oxidative Stress in Neutrophil during BBB Injury

Neutrophils, the hallmark of acute inflammation, are phagocytic immune cells found in the bloodstream. During the acute phase of inflammation, neutrophils transmigrate to the site of injury within minutes in response to chemical signals, such as IL-8, complement component 5a, N-formyl methionyl-leucyl-phenylalanine, and H_2_O_2_, in a process called chemotaxis [87,88]. In the ischemic brain, neutrophils perform several functions, including ROS production, phagocytosis, degranulation, and release of neutrophil extracellular traps (NETs), resulting in increased BBB permeability [89,90].

There are various sources of ROS in neutrophils, including NOSs and NOXs, and NOX2 may be the most important source of ROS [91]. The binding of corresponding ligands to some members of G protein-coupled receptors (GPCRs) can transform neutrophils into a “primed” state for robust activation of the NOX2 complex, as shown in Figure 2 [87]. Signals from cytokine receptors, such as TNF receptors (TNFRs) and TLRs, can also prime neutrophils, inducing phosphorylation of p47^phox^ [92,93]. When neutrophils are activated, NOX2 is activated through the similar pathways of those described previously in astrocytes, and the activated NOX produces ROS [87].

Contrary to some GPCRs, Fc receptors and some integrin receptors can directly activate NOX2 and generate ROS [87]. As shown in Figure 1D, the ligand binding of ligands to Fc receptors leads to the phosphorylation of the immunoreceptor tyrosine-based activation motifs (ITAMs) by Src family kinases (SFKs), leading to the recruitment and the phosphorylation of the Src homology domain of spleen tyrosine kinase (Syk), which further results in activation of the SH2-domain-containing leukocyte protein of 76 kDa (SLP76) signaling complex, and consequently, NOX activation [87,94].

## 3. Role of Oxidative Stress in BBB Dysfunction

**Figure 3:**
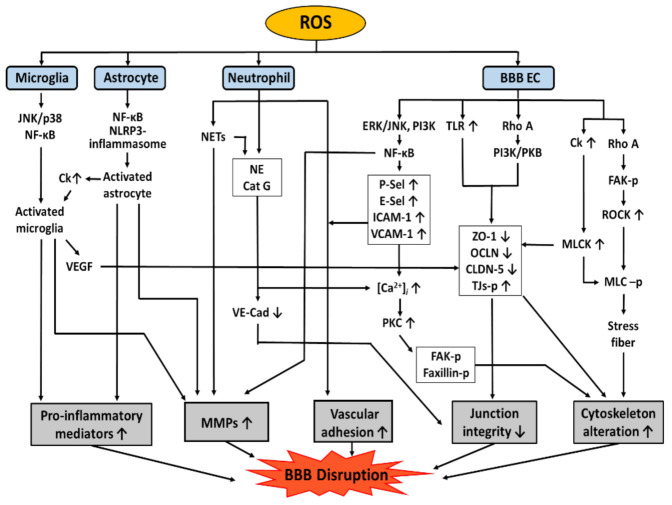
Pathways involved in ROS-induced BBB disruption. ROS can disrupt BBB by pro-inflammatory mediators, MMP activation, cytoskeleton rearrangement, and downregulation of junction integrity. Junction integrity can be downregulated by cleavage of VE-cadherin, phosphorylation, redistribution, and downregulated expression of TJ proteins. BBB disruption can also occur by cytoskeleton rearrangement caused by stress fiber formation, phosphorylation of TJ proteins, FAK, and Faxillin. Pro-inflammatory mediators can be released by adherent neutrophils, activated astrocytes and VEGF from activated microglia and activated astrocytes. MMPs can be released by NETs, activated astrocytes, VEGF, and ECs. BBB, Blood-brain barrier; Cat G, cathepsin G; Ck, chemokine; CLDN-5, cloudin-5; [Ca^2+^]_i_, intracellular calcium level; E-Sel, E-selectin; ERK, extracellular signal-regulated kinases; EC, endothelial cell; FAK, focal adhesion kinase; ICAM-1, intra-cellular adhesion molecule-1; JNK, c-Jun N-terminal kinase; MLC, myosin light chain; MLCK, MLC kinase; MMP, matrix metalloproteinase; NF-κB, nuclear factor kappa-light-chain-enhancer of activated B cells; NE, neutrophil elastase; NETs, neutrophil extracellular traps; NLRP3, nucleotide-binding oligomerization domain leucine rich repeat and pyrin do-main-containing protein 3; OCLN, occludin; p38, p38 mitogen-activated protein kinases; PI3K, phosphatidylinositol-3-kinase; P-Sel, P-selectin; PKB, protein kinase B; PKC, protein kinase C; -p, Phosphorylation; ROCK, Rho-associated protein kinase; ROS, reactive oxygen species; TJ, tight junction; TLR4, Toll like receptor 4; VCAM-1, vascular cellular adhesion molecule-1; VE-Cad, VE-cadherin; VEGF, vascular endothelial growth factor; ZO-1, zona occluden-1.

As mentioned above, oxidative stress is one of the most critical causes of BBB dysfunction [1]. A variety of pathways are involved in mediating BBB dysfunction, including modification of TJ proteins, induction of the expression of adhesion molecules (AMs), cytoskeletal reorganization, MMP activation, and NET formation and release of pro-inflammatory mediators, as shown in Figure 3. AMs are a subset of cell surface proteins that are involved in the binding of cells with other cells or with the extracellular matrix (ECM) via a process called cell adhesion [95]. There are several types of AMs on BBB ECs, such as ICAM-1, VCAM-1, platelet endothelial cell adhesion molecule-1 (PECAM), and selectins (P-Sel, E-Sel), which will be discussed in more detail in this chapter.

### 3.1. TJ Proteins

The barrier function of the BBB is mainly determined by TJ proteins, which act as gatekeepers of the paracellular space between BBB ECs, thereby governing the passage of water-soluble molecules and ions across the BBB [96]. TJs are mainly composed of three proteins: claudins, occludins, and ZO proteins [97]. Among more than 24 members of claudins (20–24-kDa), the main isoforms in brain ECs include claudin-1, -3, -5, and -12, and claudin-5 is especially known to prevent paracellular diffusion of large particles through the BBB [6]. Occludin is the main structural protein of the TJs, and its expression level can represent the structural integrity of the BBB; for instance, lower levels of occludin can reflect an increased BBB permeability. ZOs (ZO-1, ZO-2, and ZO-3) are scaffolding proteins that interact with intracellular components such as F-actin to influence cytoskeleton mobility and other functions [98]. Claudins and occludin bind to the cytoplasmic C-terminal domain of the actin cytoskeleton in BBB ECs via ZOs as accessory proteins [99].

Numerous studies have indicated a correlation between oxidative stress and alterations in TJ complexes [100]. ROS can affect TJ proteins through the following mechanisms: (1) decreased expression of TJ proteins, (2) induction of TJ proteins redistribution, and (3) phosphorylation of TJ proteins [101]. ROS increase the expression of TLRs in BBB ECs, resulting in downregulation of the expression of occludin and claudin-5 and subsequently increasing BBB permeability [102]. An exposure of BBB ECs to hypoxia reduced the expression of claudin-5, occludin, and ZO-1 [1,103]. According to Haorah J et al. [104], ONOO^−^ can induce a reduction in claudin-5 expression. Other studies have shown that LPS attenuated the expression of claudin-5 and ZO-1 in bEnd3 cells [105]. In addition to the expression level of TJ proteins, ROS can regulate the distribution of TJ proteins via the RhoA/PI3 kinase (PI3K)/protein kinase B (PKB/Akt) signaling pathway [106]. A study performed using bEnd3 has shown that the exposure to LPS changed the distribution of claudin-5 and F-actin [105]. Exposure to H_2_O_2_ leads to the redistribution of ZO-1 from the TJ sites to the cytosol, resulting in decreased transepithelial electrical resistance and increased BBB permeability [100,107]. Furthermore, ROS have also been implicated in the phosphorylation of TJ proteins that can alter their interactions with transmembrane proteins and the actin cytoskeleton, resulting in changes to the structure and barrier function of the BBB [99]. Since there are many serine/threonine and tyrosine residues that act as phosphorylation sites of TJ proteins, the states of TJ protein phosphorylation could have different effects on BBB permeability, depending on the phosphorylation type or the signaling pathway [108]. Previous research has shown that ROS induce the activation of RhoA/RhoK in human immunodeficiency virus-infected mice and, subsequently, mediate the phosphorylation of claudin-5 and occludin, leading to a decrease in BBB tightness [109]. Tyrosine phosphorylation of BBB TJ complexes induced by ROS increases intracellular gap formation and vascular permeability [99]. Changes in the phosphorylation of occludin and ZO-1 also affect the distribution of these TJ proteins [104,110].

### 3.2. AMs

While the expression of AMs is repressed under conditions not involving any type of stimulation, it is temporarily coordinated to ensure that the processes of leukocyte rolling, and firm adhesion/emigration can occur for several hours after the initiation of an inflammatory response [111]. Oxidative stress has been shown to induce the expression of AMs that affect BBB permeability by mediating leukocyte-vascular adhesion and infiltration to the brain. Therefore, inhibition of AMs expression may prevent BBB dysfunction. Oxidative stress also activates NF-κB, resulting in the expression of ICAM-1 and VCAM-1 [112], and the crosslinking of ICAM-1 activates the Ca^2+^ signaling pathways, leading to cytoskeletal alterations, thereby disrupting the BBB [113]. Thus, blocking ICAM-1 with an antibody reduces ischemic brain injury in Wistar rats [114]. Similarly, in a mouse stroke model, elimination of ICAM-1 or neutrophils reduced infarct volume, decreased mortality, and improved BBB dysfunction [115]. Other AMs, such as PECAM-1 [116], E-Sel [117], and P-Sel [118], have also been reported to be redox-regulated. ROS can increase the expression of P-Sel and E-Sel. These selectins promote neutrophil tethering and rolling adhesion by binding to their ligand P-Sel glycoprotein-1 (PSGL-1) on neutrophils [119], similar to that observed with ICAM and VCAM. In another study, stroke-induced infarct size was decreased in P-Sel knockout mice, as evidenced by BBB disruption and granulocyte infiltration [120]. This means that AMs can play a role in BBB opening, and the inhibition of AMs can prevent BBB dysfunction [121].

The receptors for AMs, such as PSGL-1, β_2_ integrin CD18, and α integrin CD11b, on neutrophils and microglia were also redox-dependent [122,123] and have been shown to participate in the initial movement of leukocytes into the ischemic region [124]. A recent report further showed that the NOX2/ROS signaling pathway can affect very late antigen-4 (VLA-4) and lymphocyte function-associated antigen 1 (LFA-1) expression in monocytes [125]. This concludes that inhibiting NOX2/ROS signaling pathways might be an effective strategy to reduce monocyte-endothelial adherence. Recruited leukocytes, such as neutrophils and macrophages, may further exacerbate injury to the BBB by producing more ROS.

### 3.3. Cytoskeletal Reorganization

Under physiological conditions, the TJ proteins (occludin and claudin) and AJ protein (cadherin) in BBB ECs are anchored to the actin cytoskeleton by multiple accessory proteins (ZO-1, ZO-2, and ZO-3), indicating that dynamic interactions between the junctional proteins (JPs) and cytoskeleton are essential for the maintenance of BBB integrity [2]. While actin is normally organized at the periphery of ECs, it reorganizes into cytoplasmic filaments called stress fibers upon encountering pathological stimuli, leading to an increase in BBB permeability [126].

Cytoskeletons of BBB ECs are known to be altered by oxidative stress via various pathways. One is the Rho-dependent pathway [106]; once elevated level of superoxide activates the Rho-dependent pathway, Rho can phosphorylate several proteins, including focal adhesion kinase (FAK) [106,127,128], mammalian diaphanous-related formin, and Rho-associated protein kinase (ROCK) [106,129]. Activated ROCK then increases phosphorylation of the myosin light chain (MLC) by either directly or indirectly inhibiting MLC phosphatases [129]. Oxidative stress can also increase the expression of chemokine receptor type 5, which subsequently activates MLC phosphorylation by MLC kinase (MLCK), leading to rearrangement of the actin structure [130]. In addition, MLCK can also phosphorylate TJ proteins, increasing the cytoskeleton reorganization. The second is the RhoA/PI3K pathway; oxidative stress selectively triggers signaling cascades, including RhoA, PI3K, and PKB/Akt. As a result, the actin cytoskeleton is rearranged, while occludin and claudin 5 are spatially distributed and disappear [106]. The third is a PKC-dependent pathway. Oxidative stress induces an increase in the expression of adhesion molecules such as ICAM-1 and VCAM-1, which can elevate intracellular Ca^2+^, and subsequently leads to PKC activation and phosphorylation of FAK and paxillin [113,131]. As a result, these pathways induce stress fiber formation, which reduces BBB integrity.

### 3.4. MMPs

MMPs are zinc-dependent endopeptidases that degrade TJ proteins and the ECM. They are usually activated by cleavage induced by other MMPs or proteases [132,133]. Physiologically, MMP expression is very low in the brain; however, it is rapidly upregulated during injury [134]. The proteolytic activity of MMPs has been demonstrated to induce BBB disruption, and therefore, the inhibition of MMP activity by tissue inhibitors of MMPs (TIMPs) may prevent BBB dysfunction [132,135]. MMP levels are increased by ROS in BBB ECs as well as in other cells of the neurovascular unit such as neurons, astrocytes, and microglia under inflammatory conditions [134]. ROS regulate the activity of MMPs through oxidation or S-nitrosylation of MMPs, leading to the subsequent activation of MMPs [136,137]. Additionally, MMP expression can be induced by ROS via ERK/c-Jun N-terminal kinase activation and NF-κB pathways [138]. Expression of MMP-1, MMP-2, and MMP-9 can also be increased by protein tyrosine kinase (PTK), which is activated by ROS. Furthermore, the levels of TIMP-1 and -2 in human BBB ECs were reduced by ROS in a PTK-dependent manner [99]. Increased activity of MMPs degrades the basement membrane and disrupts TJ assembly, resulting in increased BBB permeability [139,140]. Recent research has shown that the activation of MMP leads to cleavage of TJ proteins, which leads to detachment of ECs from the ECM and, thus, BBB disruption [141]. In addition, activation of MMP-2 and MMP-9 is also associated with BBB disruption characterized by leukocyte infiltration and microglial activation [142]

Oxidative stress-induced production of cytokines, such as IL-1β and TNF-α, can activate signaling pathways, including ERK, P38, and PI3K, increasing the binding activity of NF-κB to the promoter region of MMP-9 and, thereby, inducing the transcription of MMP-9 [143]. In mice exposed to prolonged hypoxia, the activation of MMP-9 and subsequent decrease in the expression of occludin and ZO-1, resulting in BBB permeability, has been observed [144]. Overexpression of MMP-9 is reported to cause significant degradation of occludin and claudin-5 [145]. MMP-2, which is constitutively expressed by astrocytes, provides the basis for the first local event of proteolysis, leading to basal lamina degradation and damage to BBB [134]. MMP-2 also degrades occludin and claudin-5 in a rat model of ischemic stroke [132]. Recent research has shown that MMP-3 can increase BBB permeability, and this effect may be at least partially mediated by the ERK pathway and ERK-induced disruption of inter-EC junctions (ZO-1, claudin-5, and occludin) [146].

### 3.5. NETs and Pro-Inflammatory Mediators

As explained above, neutrophils can be recruited by oxidative stress, and they adhere to activated vasculature, via the interaction of ICAM-2 and LFA-1, which can trigger the generation of ROS [131,147]. ROS generated from extravasated granulocytes can induce lipid peroxidation, which preferentially oxidizes polyunsaturated fatty acids. Indeed, since lipids are a major component of cell membranes and play a critical role in maintaining cell integrity, excessive lipid peroxidation can cause irreversible changes in the physical structure of cell membranes, which may result in BBB disruption and cell death [148,149,150]. During the acute phase of neuroinflammation, neutrophils transmigrate into the injury site by releasing granular contents, such as MMP and elastase [132,151]. For instance, TNF-α stimulates neutrophils to release elastase and cathepsin G, which can cleave VE-cadherin and damage junctional integrity [152]. Other permeability-increasing cytokines include IL-1β and chemokines (CXCL1, 2, 3, and 8) [153,154]. In addition, NETs have recently been highlighted as BBB-altering factors. NETs are web-like structures of extracellular fibers, composed of DNA produced by neutrophils, which primarily bind to pathogens [155]. NETs can effectively trap invading pathogens and disrupt their virulence by presenting a high concentration of local antimicrobial peptides, such as cathelicidins [156,157] and proteases (myeloperoxidase and neutrophil elastase) [158]. NETs are also reported to finally damage the BBB and cause harm to adjacent neurons and other brain cells [155]. In a mechanistic study, phorbol 12-myristate 13-acetate (PMA)-induced NOX have been reported to activate ERK and p38 MAPK, which result in the release of NETs from neutrophils, suggesting the involvement of ROS in NET formation [159]. The role of ROS in NET formation is supported by studies in patients with AD, which demonstrated that Aβ-induced NOX is associated with NET development and BBB disruption. Intravascular NETs can also trigger the coagulation cascade and promote thrombosis, thereby exacerbating cerebrovascular diseases [160]. Furthermore, intravascular NETs release proteolytic proteins, such as cathepsin G and MMPs, which are associated with the disruption of junctional complexes and retraction of endothelial cells.

In addition to intravascular neutrophils, extravasated granulocytes can also release NETs in the perivascular spaces as well as the brain parenchyma, which in turn can lead to microglial activation and neurotoxicity [160]. Furthermore, it is suggested that pro-inflammatory mediators released by microglial cells, such as TNF-α, IL-1, and IL-8, can induce activation of neutrophils to enhance NET release, which further activates microglial cells [151]. LPS-activated microglia induce mRNA expression as well as secretion of pro-inflammatory mediators, such as TNF-α, IL-1α, IL-1β, and IL-6 [161]. These pro-inflammatory cytokines, together with NO, further lead to increased permeability of BBB ECs and upregulation of AMs, facilitating leukocyte recruitment into the brain, and consequently, leading to brain injury [162]. Activated microglia can also induce upregulation of vascular endothelial growth factor (VEGF) [163], which renders the BBB partially open and causes leakage of serum proteins, such as fibrinogen and albumin from the blood stream into the brain parenchyma [164]. Microglia are attracted to these proteins, and perivascular microglia migrate to the damaged blood vessels, causing further damage by phagocytosis of ECs [165]. VEGF has been found to change the distribution, expression, and phosphorylation of TJ proteins such as ZO-1 and occludin [166,167], and thereby, to enhance the permeability of BBB ECs [168].

Similar to microglia, astrocytes can also be activated by ROS to induce BBB disruption. In RBA-1 cells, LPS induces phosphorylation of NF-κB p65 via an increase in the production of ROS mediated via NOX activation, which leads to increased MMP-9 mRNA and protein levels [57]. Another study in astrocytes reported that mitochondrial ROS mediates the classical NLRP3 inflammasome activation, which cleaves pro-inflammatory cytokines induced by LPS [169]. DAMPs and HMGB1 bind to receptors such as TLR4 or RAGE on astrocytes, subsequently activating astrocytes. Activated astrocytes can secrete TNF-α, IL-1β, IL-6, MMPs, and chemokines, which may indirectly and directly impact BBB integrity by disrupting claudin-5 and occludin, thereby inducing the breakdown of the BBB and immune cell infiltration [170,171]

## 4. Effects of Natural Polyphenols on BBB Dysfunction

Natural polyphenols are secondary metabolites that are produced by plants to protect themselves from other organisms. Polyphenols demonstrate a “typical polyphenol structure” (i.e., several hydroxyl groups on aromatic rings) and are classified as flavonoids (flavonols, flavones, flavanols, flavanones, isoflavones, and anthocyanins) and non-flavonoids (stilbenes, phenolic acids lignans, tannins, and hydroxycinnamic acids) [12,172]. Polyphenols have been reported to be strong antioxidants that can neutralize ROS by donating an electron or hydrogen atom, or suppress the generation of ROS by inhibiting the formation of ROS. They also act as direct radical scavengers of the lipid peroxidation chain reactions. As natural antioxidants, various polyphenols have recently been found to exert beneficial effects on neurodegenerative diseases by ameliorating BBB destruction [173]. In this chapter, we summarize the effects of two subgroups of polyphenols, namely flavonoids and non-flavonoids, on BBB dysfunction.

### 4.1. Flavonoids

Flavonoids are phenolic compounds found in various vegetables, fruits, seeds, nuts, grains, spices, wine, and tea [174,175]. They contribute to colors of the flowers and reduce the stress response in plants by scavenging ROS and UV absorption [176]. Moreover, they can exhibit pharmacological activities, such as antioxidant, anti-inflammatory, and anticancer effects [177]. Flavonoids are subdivided into subgroups such as flavonols, flavones, flavanols, flavanones, isoflavones, and chalcones [178]. This review summarizes the effects of various flavonoids on BBB dysfunction and the underlying mechanisms (Table 1).

#### 4.1.1. Flavonols-Quercetin/Kaempferol/Rutin

Numerous studies performed using various experimental models have reported that flavonols such as quercetin, kaempferol, and rutin ameliorate BBB disruption [180,184,185].

Quercetin can prevent BBB damage by regulating several ROS production pathways. In fibrillar Aβ-induced BBB damage in human BBB EC, quercetin not only prevented BBB disruption but also attenuated increased BBB permeability by preventing the overproduction of ROS and maintaining SOD activity [179]. In a rat model of cerebral I/R injury, quercetin attenuated the increased BBB permeability, upregulated the expression of tight junctions, such as claudin-5 and ZO-1, and inhibited MMP-9 expression, which is associated with the Wnt/β-catenin signaling pathway, thereby reducing brain edema and ameliorating BBB dysfunction [180]. In female rats with oxidative stress induced by polychlorinated biphenyl, the administration of quercetin exerted protective effects on the BBB by upregulating the mRNA expression of transmembrane TJ proteins (occludin, claudin-5, and JAM) and cytoplasmic accessory TJ proteins (ZO-1, ZO-2) [181]. In murine bEnd3 cells under high glucose conditions, quercetin showed antioxidant effects by reducing ROS generation and increasing relative gene expression of Nrf2 and antioxidant enzymes, including MnSOD and HO-1 [182]. Under the same conditions, quercetin ameliorates BBB disruption by reducing BBB permeability, increasing the expression of claudin-5 and inhibiting the expression of NF-κB, iNOS, TNF-α, and IL-6. Quercetin also inhibits monocyte adhesion and transmigration on cerebral ECs, and the underlying mechanisms are associated with the inhibition of E-selectin [183].

Kaempferol attenuates LPS-induced BBB dysfunction by inhibiting the increase of BBB permeability and the degradation of TJ proteins such as occludin-1, claudin-1, and CX-43 in mice [184].

Rutin has been reported to exhibit protective effects in relation to antioxidant enzymes, such as SOD in ischemic tissue injury [198]. In an in vivo rat stroke model, rutin was reported to exert a protective effect on BBB dysfunction against cerebral ischemic injury by ameliorating BBB permeability and MMP-9 activity [185].

Therefore, it can be suggested that quercetin, kaempferol, and rutin can act as protective agents against BBB dysfunction-associated diseases.

#### 4.1.2. Flavones-Baicalein/Vitexin/Luteolin

Baicalein, luteolin, and vitexin are flavones that were reported to ameliorate BBB dysfunction under oxidative stress [187,189,199].

In the human BBB EC model, baicalein, which is a known LOX inhibitor, attenuated H_2_O_2_-induced cell injury and 12/15-LOX activity. In the mouse ischemia model, baicalein reduced brain edema and BBB permeability by inhibiting the degradation of claudin-5. Therefore, it can be suggested that baicalein protects the brain from oxidative stress via the inhibition of 12/15-LOX [186]. In another rat intracerebral hemorrhage (ICH) model, baicalein contributed to the reduction of brain edema and BBB permeability and increasing ZO-1 protein levels, and also inhibited MAPK and NF-κB signaling pathways, leading to the downregulation of iNOS and ONOO^−^ production and preventing BBB disruption. Furthermore, baicalein inhibited cell apoptosis by reducing the level of caspase-3 protein [187].

In an in vitro Aβ-induced BBB damage model, luteolin protected human BBB ECs and human astrocytes by improving cell viability and ameliorated BBB dysfunction by reducing COX-2 expression. These effects may be attributed to the inhibition of p38 MAPK activation and the NF-κB signaling pathway. Furthermore, luteolin exhibited a slight decrease in ROS generation [188]. Moreover, in another in vivo model of Aβ damage, luteolin was shown to maintain BBB integrity by ROS scavenging and redox balancing [200].

A study of hypoxic injury showed that vitexin ameliorated BBB dysfunction by increasing cell viability and reducing BBB permeability. The underlying mechanism for improving BBB permeability includes upregulation of the expression of TJ proteins (ZO-1 and claudin-5) and downregulation of the expression of MMP-2 and MMP-9. Furthermore, vitexin could reduce ONOO^−^ generation and NO level by inhibiting iNOS activity. Vitexin also increased eNOS activity through the PI3K/Akt pathway [189].

#### 4.1.3. Flavanols-Catechin/EGCG/Theaflavin

Flavonols, including catechin, epigallocatechin gallate (EGCG), and theaflavin, have been demonstrated to attenuate BBB dysfunction under oxidative stress [190,191,192].

In an in vivo rat model of traumatic brain injury, catechin attenuated the increase in BBB leakage by inhibiting the loss of TJ proteins by upregulating the mRNA levels of ZO-1 and occludin. It also reduced the brain water content and infarct volume [190].

In the LPS-induced human BBB EC damage model, EGCG attenuated the increase in BBB leakage and the loss of TJ proteins, such as claudin-5 and occludin, by upregulating their mRNA levels. In addition, EGCG inhibited the increase in the expression of adhesion molecule (ICAM-1, VCAM-1) expression by downregulating their mRNA levels and attenuating monocyte adhesion on human BBB EC [191].

In a rat ICH model, theaflavin ameliorated the increase in BBB leakage, brain edema volume, and oxidative stress by scavenging ROS [192].

#### 4.1.4. Flavanones-Pinocembrin/Hesperidin

Flavanones are a class of flavonoids that include pinocembrin and hesperidin. They can attenuate BBB dysfunction under oxidative stress [194,195].

In an in vivo study involving the global cerebral I/R(CI/R) rat model, pinocembrin attenuated BBB dysfunction by inhibiting the increase in BBB permeability and reducing the development of brain edema [193]. In a rat model of cerebral ischemia, pinocembrin exerted protective effects on cerebrovascular units by maintaining the integrity of the BBB and increasing the mRNA levels of TJ proteins, such as occludin and ZO-1. In addition, pinocembrin also inhibited the activation of microglia and astrocytes and the initial leukocyte migration by downregulating the expression of adhesion molecules, such as ICAM-1 and VCAM-1. Furthermore, pinocembrin reduced the expression of MMP-9. The inhibition of MMP-9 expression may be due to a reduction in ROS levels [194].

Hesperidin ameliorated brain edema in a mouse stroke model. Hesperidin also inhibited BBB disruption by inhibiting the degradation of claudin-5 and the redistribution of ZO-1 in mouse brain and bEnd3 cells. In bEnd3 cells, hesperidin exerted antioxidative effects by scavenging ROS and inhibiting FoxO3a translocation, which was reported to regulate the expression of genes related to apoptosis and oxidative stress. Therefore, ROS can contribute to the regulation of BBB integrity as an upstream signaling pathway for FoxO3a/MMP3 and MMP9-mediated degradation of claudin-5 and redistribution of ZO-1 [195].

#### 4.1.5. Isoflavones and Chalcones-Puerarin/Isoliquiritigenin

Other flavonoid antioxidants include puerarin and isoliquiritigenin, which can also be characterized as isoflavones and chalcones [197,201].

In the subarachnoid hemorrhage mouse model, puerarin protected the brain by attenuating the increase in BBB permeability and brain edema volume. In addition, puerarin exerts antioxidant effects by reducing ROS and activating antioxidative proteins, such as SOD2 and SIRT3 [196].

In a rat model of ICH, isoliquiritigenin protected the brain by reducing brain edema and attenuating BBB dysfunction by inhibiting the increase in BBB permeability and exerting antioxidant effects by reducing ROS levels and increasing Nrf2 levels and antioxidant enzyme activities, such as SOD and HO-1. In addition, isoliquiritigenin reduced the infiltration of neutrophils and the recruitment of microglia to the injury site of the brain, consequently inhibiting brain edema [197].

### 4.2. Non-Flavonoid Polyphenols

As non-flavonoid polyphenols, stilbenes and phenolic acids have been found in various plants [178,202]. They have been reported to exhibit pharmacological activities, such as antioxidant and anti-inflammatory effects [203,204]. In this section, we focus on the effects of non-flavonoid polyphenols including resveratrol, caffeic acid, and gallic acid on BBB dysfunction and the underlying mechanisms (Table 2).

#### 4.2.1. Stilbenes-Resveratrol

Stilbenes are polyphenols that have defensive effects, such as antifungal phytoalexins against infection or injury in many plant species [202]. A well-known stilbene, resveratrol is found in grapes, red wine, berries, knotweed, peanuts, and other plants, and is reported to have anticancer, anti-inflammatory, and antioxidant properties [205]. Furthermore, numerous studies have reported that resveratrol exerts protective effects on the BBB [206,207].

Resveratrol protected ECs by reducing oxidative stress and inhibiting the release of VEGF. VEGF is a mediator that can impact the integrity of the BBB. Moreover, resveratrol attenuated monocyte adhesion on human BBB EC and inhibited the expression of adhesion molecules, such as PECAM and VCAM-1 [208]. Under condition of high glucose levels, resveratrol inhibited cell apoptosis and attenuated the increase in ROS levels by inhibiting the activation of NOX1 and the increase of *NOX1* mRNA via the inhibition of NF-κB activation in bEnd3 cells [209]. In a mouse model of aging, resveratrol protected the brain by reducing ROS levels and the mRNA expression of *NOX1, NOX2,* and *NOX4*. Resveratrol exerts antioxidant activity by reducing ROS levels in both astrocytes and BBB ECs, whereas its inhibitory effects on mRNA of *NOX2* and *NOX4* were shown in BBB ECs [210].

An in vitro study of BBB ECs further showed that resveratrol increased cell viability and ameliorated BBB dysfunction by reducing BBB permeability and ROS levels and inhibiting oxLDL-induced disruption of TJs, such as occludin and ZO-1. Resveratrol also improved the disruption of F-actin and cytoskeleton and attenuated cerebral endothelial cell apoptosis by reducing cytochrome C release and caspase-3 and -9 activation and by regulating the Bcl-2/Bax ratio [207]. In a mouse model of autoimmune encephalomyelitis, resveratrol protected the BBB by inhibiting BBB leakage and the loss of TJs, such as ZO-1, claudin-5, and occludin, as well as the expression of AMs, such as ICAM-1 and VCAM-1. In addition, resveratrol exhibited antioxidant properties by reducing the mRNA levels of *NOX2* and *NOX4* [206].

**Table 2:** Effects of non-flavonoid polyphenols on oxidative stress-induced BBB dysfunction.

Antioxidants		Model	Insult	Findings	Reference
stilbenes	resveratrol	HCMEC	CSE	VEGF↓, PECAM↓, VCAM-1↓, monocyte adhesion↓	[208]
		bEnd3	high glucose	cell apoptosis↓, NF-kB↓, NOX1↓, ROS↓	[209]
		mouse aged	aging	cortical tissue: ROS↓, NOX1↓, NOX2↓, NOX4↓	[210]
		rat aged	aging	CMVEC: ROS↓, NOX2↓, NOX4↓,	
				astrocyte: ROS↓	
		mouse cEC	oxLDL	BBB permeability↓, occludin↑, ZO-1↑, cell viability↑ ROS↓, cytC↓, F-actin↑, Bcl-2/Bax ratio↑, caspase-3↓, caspase-9↓, apoptosis↓	[207]
		mouse + EAE	MOG	BBB permeability↓, occludin↑, ZO-1↑, claudin-5↑, ICAM-1↓, VCAM-1↓, iNOS↓, NOX2↓, NOX4↓	[206]
phenolic acid	caffeic acid	bEnd3	high glucose	NF-kB↓, ROS↓, NOX4↓ Nrf2↑	[211]
		mouse CI/R	MCAO	VE-cadherin↑, MPO↓, infarct volume↓	
		bEnd3	high glucose	NF-kB↓, ROS↓, NOX4↓, Cu/ZnSOD↑, Nrf2↑	[182]
		bEnd3	high glucose	claudin-5↑, occludin↑, ZO-1↑, ZO-2↑, NF-kB↓, COX-2↓,	[183]
	gallic acid	rat CI/R	4VO-I/R	BBB permeability↓, lipid peroxydation↓, SOD↑	[212]
		rat CI/R	4VO-I/R + DPM	lipid peroxydation↓, SOD↑	
		bEnd3	high glucose	IL-6↓, ROS↓, NOX4↓, Cu/ZnSOD↑, Nrf2↑	[182]
		bEnd3	high glucose	claudin-5↑, occludin↑, ZO-2↑, NF-kB↓, iNOS↓	[183]

4VO, 4-vessel transient occlusion; BBB, blood-brain-barrier; Bcl-2/Bax, B-cell lymphoma-2/-associated; bEnd3, mouse brain endothelial cell; cEC, Cerebrovascular endothelial cell; CI/R, cerebral ischemia/reperfusion; CMVEC, cerebromicrovascular endothelial cell; COX-2, cyclooxygenase-2; CSE, cigarette smoke extract; Cu/ZnSOD, copper-zinc superoxide dismutase; cytC, cytochrome C; DPM, diesel particular matter; EAE, enchephalomyelitis; HCMEC, human cerebral microvascular endothelial cell; I/R, ischemia/reperfusion; ICAM-1, intercellular adhesion molecule-1; MCAO, middle cerebral artery occlusion; MOG, myelin oligodendroglial glycoprotein; MPO, myeloperoxidase; NF-kB, nuclear factor kappa-light-chain-enhancer of activated B cells; NOS, nitric oxide synthase; NOX, NADPH oxidase; Nrf2, nuclear respiratory factor 2; oxLDL, oxidized LDL; PECAM, platelet endothelial cell adhesion molecule; ROS, reactive oxygen species; SOD, superoxide dismutase; VCAM-1, vascular cell adhesion molecule-1; VE, vascular endothelial; VEGF, vascular endothelial growth factor; ZO, zonula occludin.

#### 4.2.2. Phenolic Acid-Caffeic Acid/Gallic Acid

Caffeic acid and gallic acid are phenolic acids present in many fruits and vegetables. These compounds exert beneficial effects, such as antioxidant effects [204]. Furthermore, they can attenuate BBB dysfunction under conditions involving oxidative stress [182,183].

In an in vitro study of bEnd3 cells, high glucose induced a decrease in *Nrf2* gene expression and following elevation in ROS level [211]. In contrast, Nrf2 expression levels were increased in an in vivo hyperglycemic model. This discrepancy in Nrf2 expression in vivo and in vitro may be explained by various cell populations in brain tissue, and different durations of hyperglycemic condition. Caffeic acid abolished these effects of high glucose on Nrf2 expression and ROS level [211].

In a rat model of cerebral IR, gallic acid attenuated oxidative stress by upregulating the activity of SOD, downregulating lipid peroxidation and reducing BBB permeability, thereby preventing BBB disruption. However, there was no effect on BBB permeability when rats were subjected to I/R following exposure to air pollution, which may be due to the severity of the damage [212].

Caffeic acid, in particular, also inhibits NF-κB [182]. They also improved gene expression of TJ proteins, such as claudin-5, occludin, ZO-2; and inhibited NF-κB activation [183]. Caffeic acid particularly increased ZO-1 protein expression. In addition, the phenolic acids ameliorated BBB disruption by attenuating an increase in BBB permeability. Moreover, these compounds inhibited monocyte chemoattractant protein-1 (MCP-1) expression, suggesting that these compounds can inhibit monocyte adhesion and transmigration in BBB ECs [183].

## 5. Conclusions

The BBB plays an important role in maintaining the homeostatic balance between brain parenchyma and systemic circulation under physiological conditions, whereas it can be disrupted under pathological conditions. Accumulating evidence shows that BBB disruption is a crucial process in various neuroinflammatory diseases such as stroke and AD. Since BBB dysfunction has been reported to be closely related to oxidative stress, targeting oxidative stress and BBB disruption may be a promising therapeutic strategy for neuroinflammatory diseases.

In this review, we focused on the understanding of the pathways involved in the generation of oxidative stress in cells that constitute or surround the BBB under pathological conditions. We also showed that ROS can trigger a variety of signaling pathways in cells associated with the BBB, leading to TJ activation, AJ modification, cytoskeletal reorganization, and MMP activation. All of these pathological processes result in BBB dysfunction and eventually neuroinflammatory diseases. Therefore, antioxidants are likely to act as potential protective agents against BBB-related diseases. Various natural polyphenols have been reported to exert beneficial anti-oxidant effects on BBB disruption. In addition, we summarized the potential of natural polyphenols against oxidative stress and BBB disruptive pathology. In conclusion, this review suggests that a number of natural polyphenols can act as promising neuroprotective agents, especially for BBB dysfunction-related brain diseases.

## Figures and Tables

**Table 1:** Effects of flavonoids on oxidative stress-induced BBB dysfunction.

Antioxidants		Model (Animal/Cell)	Insult	Findings	Reference
flavonol	quercetin	HBMEC	Aβ_1-40_	BBB permeability↓, ROS↓, SOD↑	[179]
		rat CI/R	BCCAO	BBB permeability↓, claudin-5↑, ZO-1↑, MMP-9↓, brain edema↓, β-catenin↑	[180]
		rat	PCB	occludin↑, claudin-5↑, JAM↑, ZO-1↑, ZO-2↑,	[181]
		bEnd3	high glucose	NF-kB↓, ROS↓, HO-1↑, MnSOD↑	[182]
		bEnd3	high glucose	BBB permeability↓, claudin-5↑, MCP-1↓, E-selectin↓, monocyte adhesion↓, NF-kB↓, iNOS↓,	[183]
	kaempferol	Mouse	LPS	BBB permeability↓, claudin-1↑, occludin↑, CX43↑, MCP-1↓, COX-2↓, iNOS↓,	[184]
	rutin	rat CI	photothrombosis	BBB permeability↓, MMP-9↓, SOD↑	[185]
flavone	baicalein	HMBEC	H_2_O_2_	cell injury↓	[186]
		rat CI	MCAO	claudin-5↑, BBB permeability↓, brain edema↓	
		rat ICH	collagenase (IV)	ZO-1↑, BBB permeability↓, ONOO^−^↓, iNOS↓, brain edema↓, NF-kB↓	[187]
	luteolin	HBMEC	Aβ_1-40_	BBB permeability↓, cell viability↑, NF-kB↓, p38-MAPK↓, COX-2↓, ROS↓	[188]
	vitexin	HBMEC	OGD/R	claudin-5↑, ZO-1↑, MMP-9↓, MMP-2↓, BBB permeability↓, caspase-3↓, ONOO-↓, iNOS↓, eNOS↑	[189]
flavanols	catechin	rat TBI	CCI	BBB permeability↓, occludin↑, ZO-1↑, iNOS↓, brain edema↓, infarct volume↓	[190]
	(-)-EGCG	HCMEC	LPS	BBB permeability↓, claudin-5↑, occludin↑, ICAM-1↓, VCAM-1↓, monocyte adhesion↓, NF-kB↓	[191]
	theaflavin	rat ICH	collagenase (VII)	BBB permeability↓, ROS↓, brain edema↓, CXCL1↓, caspase-1↓, NF-kB↓	[192]
flavanones	pinocembrin	rat CI/R	OGD/R	BBB permeability↓, brain edema↓	[193]
		rat CI	MCAO	occludin↑, ZO-1↑, MMP-9↓, ICAM-1↓, VCAM-1↓, iNOS↓, astrocyte activation	[194]
	hesperidin	mouse CI	MCAO	BBB permeability↓, claudin-5↑, ZO-1↑, brain edema↓	[195]
		bEnd3	N_2_ gas	BBB permeability↓, claudin-5↑, ZO-1↑, MMP-3↓, MMP-9↓, ROS↓, FoxO3a↓	
isoflavone	puerarin	mouse SAH	endovascular perforation	BBB permeability↓, brain edema↓, ROS↓, SIRT3↑, SOD2↑	[196]
chalcone	isoliquiritigenin	rat ICH	collagenase (VI)	BBB leakage↓, ROS↓, Nrf2↑, SOD↑, HO-1↑, brain edema↓, NF-kB↓	[197]

Aβ, amyloid beta; BBB, blood-brain barrier; ROS, reactive oxygen species; BCCAO, bilateral common carotid artery occlusion; Bcl-2/Bax, B-cell lymphoma-2/-associated X; bEnd3, mouse brain endothelial cell; CCI, controlled cortical impact; CI/R, cerebral ischemia/reperfusion; CI, cerebral ischemia; COX-2, cyclooxygenase-2; CX43, connexin 43; CXCL, C-X-C motif chemokine ligand; EGCG, epigallocatechin-3-gallate; eNOS; endothelial nitric oxide synthase; FoxO3a, Forkhead box O 3a; GSH, glutathione; hAs, human astrocyte; HBMEC, human brain microvascular endothelial cells; HCMEC, human cerebral microvascular endothelial cell; HO-1, heme oxygenase-1; ICAM-1, intercellular adhesion molecule 1; ICH, intracerebral hemorrhage; iNOS, inducible nitric oxide synthase; JAM, junctional adhesion molecules; LPS, lipopolysaccharide; MAPK, mitogen-activated protein kinases; MCAO, middle cerebral artery occlusion; MCP-1, monocyte chemoattractant protein1; MMP, matrix metalloproteinase; MMP9, Matrix metalloproteinase 9; MnSOD, manganese superoxide dismutase; NF-kB, nuclear factor kappa-light-chain-enhancer of activated B cells; OGD/R, oxygen and glucose deprivation/reoxygenation; PCB, polychlorinated biphenyl; SAH, subarachnoid hemorrhage mice SIRT3, Sirtuin 3; SOD, superoxide dismutase; TBI, traumatic brain injury; VCAM-1, vascular cell adhesion molecules; ZO, zonula occludens.
